# Automatic Detection of Group Recumbency in Pigs via AI-Supported Camera Systems

**DOI:** 10.3390/ani13132205

**Published:** 2023-07-05

**Authors:** Alexander Kühnemund, Sven Götz, Guido Recke

**Affiliations:** 1 Hochschule Osnabrück, Fachbereich Landwirtschaftliche Betriebswirtschaftslehre, Oldenburger Landstraße 24, 49090 Osnabrück, Germany; 2 VetVise GmbH, Bünteweg 2, 30559 Hannover, Germany; sven.goetz@vetvise.com

**Keywords:** artificial intelligence, animal welfare, animal behavior, automatic monitoring

## Abstract

**Simple Summary:**

For this study, several pens of weaned piglets were recorded with cameras on a commercial farm. The goal was to use velocity data to establish an automated method of identifying when all animals are lying down. This automated method had an accuracy of 94.1%. This method can benefit modern farm management and highlight otherwise overlooked conditions in the barn.

**Abstract:**

The resting behavior of rearing pigs provides information about their perception of the current temperature. A pen that is too cold or too warm can impact the well-being of the animals as well as their physical development. Previous studies that have automatically recorded animal behavior often utilized body posture. However, this method is error-prone because hidden animals (so-called false positives) strongly influence the results. In the present study, a method was developed for the automated identification of time periods in which all pigs are lying down using video recordings (an AI-supported camera system). We used velocity data (measured by the camera) of pigs in the pen to identify these periods. To determine the threshold value for images with the highest probability of containing only recumbent pigs, a dataset with 9634 images and velocity values was used. The resulting velocity threshold (0.0006020622 m/s) yielded an accuracy of 94.1%. Analysis of the testing dataset revealed that recumbent pigs were correctly identified based on velocity values derived from video recordings. This represents an advance toward automated detection from the previous manual detection method.

## 1. Introduction

Recent scientific and technological developments yielded substantial opportunities for the use of AI-based surveillance technology to monitor animals on large-scale farms [1]. Opportunities for improvement range from animal welfare to economic benefits for farmers. In times of resource scarcity and energy crisis, improved management is essential to operate the business in an economically sustainable manner. Various approaches have already been used to monitor pigs with this technology. The method has been used to assess the recumbency of animals [2,3,4], estimate their weight [5] and detect a specific action such as tail biting [6]. Notably, observations of growing piglets are one of the greatest opportunities for the use of this technology.

Since the 1990s, several researchers have studied different approaches to observe and evaluate the behavior of animals [7,8,9,10]. Researchers have discussed the critical factors of time, sample size, observer influence, and consistency of results and interpretation [9,11,12]. Over time, various methods have been developed for observing animal behavior. In focal sampling, one or two animals are observed while the rest of the group is ignored. This enables precise determination of the behavior of these individual animals. However, information regarding group dynamics is lost. In contrast, scan sampling of the entire group also leads to the loss of details, as a human observer is not able to watch them all at the same time. Changing the observation period or duration provides more options to tailor the behavioral observation methods to the species. Another approach is interval sampling, which involves collecting many hours of video footage. This method allows the processing of many hours of video material at once. However, much information is lost between sampling intervals, and a consistent picture of the daily routine of the observed individuals cannot be obtained. This problem can be overcome by observing the animals continuously (continuous sampling), but this requires an enormous amount of time [7,13].

AI-based monitoring and evaluation can be used to address this problem, such as by observing resting behavior in groups of pigs. Under normal conditions, pigs spend 80–90% of the day recumbent, but they do not all rest at the same time [14,15]. Indeed, this proportion differs for estimates of times in which all pigs are recumbent. In wild pigs or those reared in alternative housing systems with straw, the rates of resting behavior are significantly decreased. For example, wild boars spend between eight and eleven hours per day foraging. These periods are interrupted by interactions with conspecifics, locomotion, and exploration of the environment or rest phases [16,17].

Simultaneous resting behavior in the whole group of pigs is an indicator of welfare under different thermal conditions [18,19] or the health status of the group [20]. Studies have investigated recumbent behavior and its relationship with behavioral factors [21]. To detect this behavior, it is necessary to observe the entire group of pigs, but this makes it difficult to automate the process and assess situations in real-time. To date, various systems can recognize recumbent pigs and the posture of the recumbent individual. [2] demonstrated the benefits of automated scoring of recumbent behavior in groups of pigs as well as the potential for automated climate adjustments reliant on this behavior [22]. Several studies have also investigated the usefulness of video technology for monitoring and interpreting lying behavior. Thus, researchers have used cameras and image processing to study resting behavior and identify behavioral changes in pigs [22,23,24,25]. New computer-vision systems can also monitor the behavior of individual pigs, including standing, sitting, and recumbent positions [26]. However, when using technology to identify recumbent pigs and piglets in a group, segmentation technologies are often used to identify pigs and evaluate their recumbent behavior. A systematic review concluded that technologies using this approach have high accuracy in segmenting pigs but are unable to detect overlapping pigs [1]. In addition, this technology overlooks pigs that move while all other observed animals are recumbent. This prevents studies from evaluating the lying behavior of the entire group [22].

To date, research has shown that the use of AI has the potential to automatically assess pig behavior and that such measures can benefit animal welfare. However, there is no reliable method that can identify instances in which all pigs in a group are recumbent. In particular, small pigs overlap with other pigs in the group when cold. Automated analysis of group recumbency using image analysis is preferable to ensure that all pigs are recumbent. Piglets are particularly sensitive to external factors during the rearing phase. Stress after weaning, new feed, movements to different groups (and associated ranking fights), new housing environments, and climatic conditions can affect the growth and development of piglets. These immature pigs are not able to protect themselves effectively from attacks or to escape from dangerous situations. For these reasons, special precautions are needed to ensure that piglets remain healthy during this vulnerable stage [27].

In our research, we used the cumulative velocity of all observed animals to detect resting behavior. The observation system defines velocity as the movement of observed pigs in meters per second. To enhance the applicability of our findings to real-world settings, the data were collected on a real farm and not generated in an experimental setting. Since the various AI-supported detection techniques provide opportunities for farm management, it is essential to create an automated system that is usable in commercial farms given the existing infrastructure. For this purpose, a simple two-dimensional camera system was paired with an object-tracking AI system, which can be used in ordinary pig farms.

Several studies have monitored pigs and piglets using similar technology with different objectives. The aim of our study was to obtain images of piglets when all animals in a group are recumbent. For this purpose, we used camera systems that perform object tracking with the help of artificial intelligence. The automatic identification of such images can help farmers recognize previously overlooked conditions and thus improve farm management. Moreover, such data are helpful for solutions that automatically interpret group resting behavior. The focus of our work was on piglets because individual recognition of recumbent piglets is often difficult in groups due to overlap. Farmers can use such images to interpret the behavior of their animals and make adjustments to housing conditions.

## 2. Materials and Methods

### 2.1. Animals

Eighteen weanling (four-week-old) pigs of the hybrid cross Tempo × BHZP Victoria were stocked in each of the 12 experimental pens. The animals were fed ad libitum and had constant access to fresh water. During the experiment, the weaners were kept under normal farm conditions and did not receive any special treatment. No actions during the study caused pain, suffering, or harm to these animals. Therefore, no additional permit was required under the Ordinance for the Protection of Animals Used for Experimental or Other Scientific Purposes.

### 2.2. Experimental Design

The animals were housed for the duration of the rearing period (postnatal weeks 4 to 10) in 12 identically furnished pens on a commercial farm. The trial was conducted under normal farm conditions. It was integrated into the farmer’s daily work in order to obtain results as close to practice as possible. However, two of the pens were excluded from the study due to suboptimal camera placement. Thus, 10 pens and 180 piglets were included in the study.

**Figure 1:**
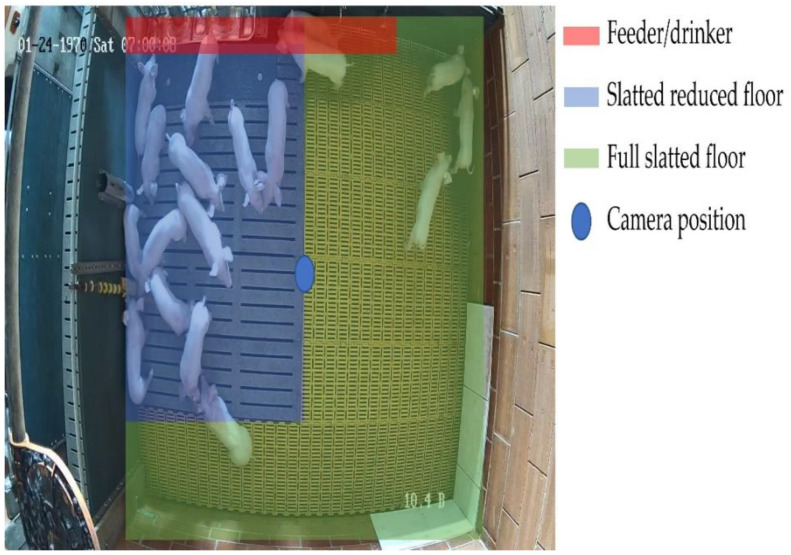
Test pen with marked camera position.

The pens were 2.55 m × 3.2 m in size (8.16 m^2^, 0.54 m^2^/animal) and included a narrow-slatted floor (yellow) in some areas and a wide-slatted floor (blue) in others (see Figure 1). The piglets had constant access to fresh water via a nipple drinker and a trough drinker (red). Feed was provided from an automatic mash feeder. Various activity materials, such as chains or wooden sticks, were also attached to the walls of the pens.

The network cameras (DS-2CD2123G2-I; 2.8 mm; Hikvision, Hangzhou, Zhejiang, China; blue dot) were mounted in the center of each pen 2.36 m above the recorded animals. This allowed us to film and analyze the entire pen but not adjacent pens (e.g., no measurements in other pens or human interaction in the data analysis). The behavior and activity of the pigs were continuously recorded (24 h/day) with cameras and stored digitally.

The recumbent behavior of animals was determined on a computer connected to the cameras with the aid of the PigBrother system (VetVise GmbH, Hannover, Germany).

### 2.3. Measurements

The animals were continuously observed with a camera system for 24 h on fourteen consecutive days. The recordings were stored and analyzed using the VetVise system.

**Figure 2:**
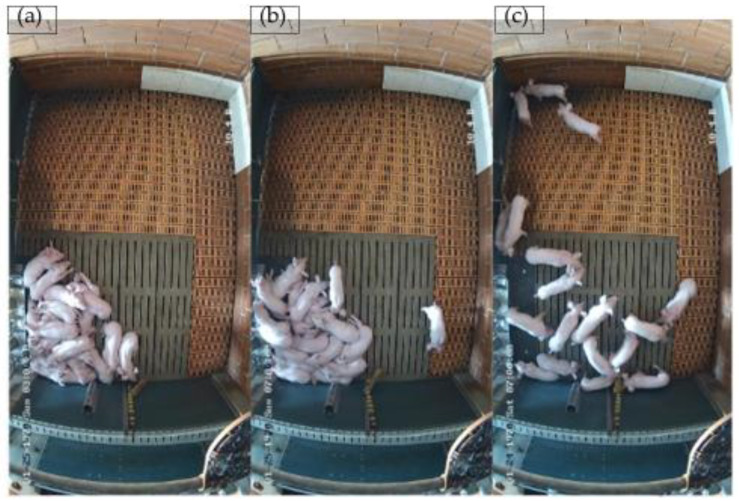
Groups of pigs that are all recumbent (**a**), mainly recumbent with a few active animals (**b**), and all active (**c**).

To evaluate resting behavior, single frames were extracted from the video recordings every 20 min, resulting in 9634 images during the experimental period. Pictures without animals were excluded. In addition, for privacy reasons, images showing people in whole or in part were not included in the analysis. The images were separately coded in a binary manner by two observers (A.K. and S.G.). The coding system is presented in Table 1. If all animals were recumbent, the image was coded as “0” (see Figure 2a). If at least one pig was in motion, i.e., not recumbent, or sitting, the image was coded as “1” (see Figure 2b,c). If pigs lay on top of each other and one pig could have been in a partially standing position, this case was coded as “0”. The results of the assessment were entered into an Excel spreadsheet for further analysis.

At the beginning of the evaluation, each observer evaluated 100 images according to the scheme described above. Images that were not uniformly labeled were discussed, and their code was decided by a majority vote. Based on these results, an observer adjustment was made to ensure that the results were comparable. After this coding pilot test, both coders received all image files and coded them independently. Of the 9634 images, 238 images were identified as having coding discrepancies after the independent coding process. These images were reviewed together and discussed, and a consensus was reached. The intercoder agreement was 97.5%.

For the next step, an indicator was developed to provide automatic detection of all recumbent animals. The movement of animals was used. The movement was defined as velocity (*v*) in meters (Δ*p*) per second (Δ*t*) and was recorded by the camera. These data represent the average sum of distance changes from one frame to the next frame for all animals detected as standing divided by the time interval in seconds.

v=Δp∕Δt

The cumulative velocity value of the group was determined by the PigBrother system from VetVise GmbH. Using artificial intelligence, PigBrother generates animal velocity values from video data and combines them in each trial. PigBrother uses an object-tracking method [28] to calculate the cumulative velocity. The objects (pigs) are identified by an artificial neural network in the image recognition process [29]. The objects recognized by the artificial neural network are then tracked by object tracking and numerical values (meters per second) are compiled. This method of object classification has been used in related approaches [30,31,32].

The movement data were collected over the entire period to identify any movements of the group of pigs. To make precise measurements regarding the amount of movement, all measurements within a 5-min interval were summarized. To select images with a high probability of containing only recumbent pigs, we analyzed the 5-min average velocity of the groups of pigs in the different pens. The “velocity values” were assigned to the previously labeled images based on their time stamp and pen name in the database, such that each labeled image was assigned to the real “velocity value”.

To obtain information about the amount of movement and to determine thresholds for automatic detection, a sample of 1023 images over different time frames from different pens was linked to the movement data. The movement data were then combined with the active (=1) and inactive (=0) image data to identify the threshold for group recumbency of pigs.

To ensure accurate prediction of whether animals were moving, the threshold for automation was defined as the mean value for recumbent animals plus the standard deviation. In the first analysis, the data showed large intervals of movement while all pigs were recumbent. Upon further examination, we found that accuracy was related to the condition of the cameras. Dirty camera lenses reduced detection efficiency. To quantify the dirtiness of cameras, a blur detection algorithm commonly used in image processing was applied using OpenCV packages (Python 3.10.7, Wilmington, DE, USA, OpenCV version 3.1.0, Mountain View, CA, USA). This algorithm evaluates the blurriness of an image by quantifying the edges in an image. For this purpose, the Laplacian is first calculated for each pixel of the image. Laplacians consider the second-order derivative of the image topology, which yields large values when there are large differences between two adjacent pixels. This means that the Laplacian of one pixel is especially large if it represents the edge of a shape in the image.

In blurred images, there are fewer edges (and fewer solid areas). These images have Laplacians with a lower variance; hence, we required large variance when selecting images that are not blurred.

The variance of the resulting values was then taken as a measure of blur. A Laplacian value greater than 1000 was set as the internal threshold for blurring, i.e., all images with a lower value were considered to be blurred and excluded from further analysis. After the application of this criterion, 3960 images remained.

### 2.4. Statistical Analyses

To examine whether there was a significant correlation between the measured velocity and the recumbency code of an image, an independent-sample *t*-test was performed. The probability of error was set at α < 0.05.

The threshold method was used to calculate the velocity value with the highest matching and lowest error rate, i.e., the highest velocity value with the fewest images labeled as standing. This method was chosen because the determined value includes as few misclassified data points as possible (e.g., a low-velocity value but an image with standing pigs).

Simple thresholding is a standard procedure in R statistical computing. First, all values are sorted in ascending order to obtain an overview of the values. After setting an initial threshold, all images coded as 1 and above the threshold were counted. Then, the threshold was increased in a stepwise manner using a loop function, and the number of images coded as 1 was counted again. The threshold value at which the number of images coded as 1 and above the threshold value no longer decreased was recorded as the highest threshold value. Subsequently, we checked whether the threshold value contained the highest velocity value in an image coded as 0. If it did not, the process was repeated by setting the initial threshold value to the highest velocity value in an image coded as 0. This method allowed us to identify the threshold with the fewest 1-coded images above the threshold and the highest velocity value in images coded as 0.

To test our data, we used R Studio, version 2022.12.0+353 (package “caret” [33]). For reproducibility, set.seed was set to set.seed(123). The procedure implements a threshold method and evaluates its performance using a 10-fold cross-validation.

In the first step, the 10-fold cross-validation is conducted to assess the model’s performance. This method divides the dataset into 10 equally sized subsets or folds. In each iteration, one fold is used as the test dataset, while the remaining 9 folds are used for training the model. This process is repeated 10 times, with each fold serving as the test dataset once.

For each iteration of cross-validation, the optimal threshold value is determined. The threshold is used to classify observations into either the positive class (movement) or the negative class (no movement). By trying out different threshold values, the one that maximizes accuracy is selected. Once the optimal threshold is obtained, predictions are made for the corresponding test dataset. The speed of each test observation is compared against the threshold to determine if movement is present or not. Subsequently, various performance metrics are computed to evaluate the model’s accuracy. Accuracy represents the proportion of correct predictions compared to the total number of predictions.

In addition, the average sensitivity (true positive rate, recall) and specificity are also calculated (see Table 2). Sensitivity measures the model’s ability to correctly identify the actual positive cases, while specificity assesses its ability to correctly identify the actual negative cases. The average sensitivity is reported as 0.978, and the average specificity is reported as 0.608.

Finally, the average values of all the performance metrics across the folds are computed. The results reveal an average accuracy of 0.9405, an average efficiency of 0.6260, an average sensitivity of 0.978, and an average specificity of 0.608. These values provide insights into the model’s performance, its ability to classify both positive and negative cases accurately, and its overall sensitivity and specificity.

## 3. Results

The blur-adjusted dataset contained 3960 images, including 3549 pictures with moving animals and 411 showing group recumbency. Most of the recorded images were coded as containing moving animals (89.62%). Table 3 displays the frequencies of the images included in the analysis according to the pen (pens 1–10) in which they were taken and the code with which they were labeled (0 = all pigs recumbent/1 = at least 1 animal standing).

The independent-sample *t*-test revealed a statistically significant difference between the measured mean values of velocity for images containing all recumbent and some moving piglets (*p* < 0.001). This allowed us to use the data for further analysis.

**Figure 3:**
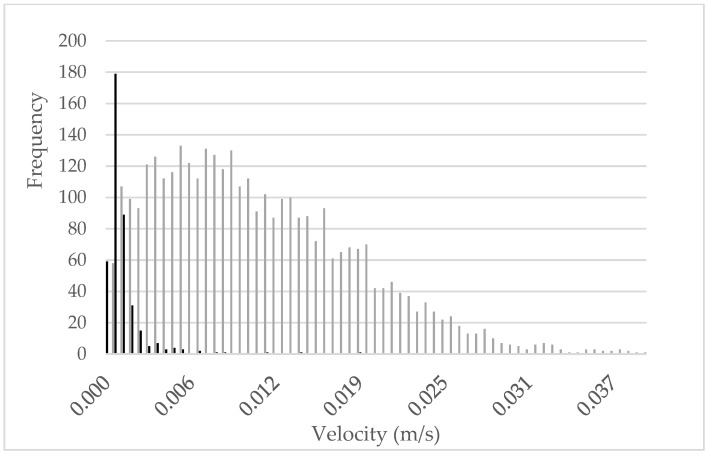
Histogram of movement velocity of recumbent pigs (black) and moving pigs (gray).

Figure 3 shows the frequencies of average pig velocities for images in which all pigs are moving (gray) or at least one pig is recumbent (black). As can be observed, images of recumbent pigs were associated with lower velocities. This indicates that the assumption that low velocities are associated with group recumbency is suitable for detecting this behavior in weaners.

Our threshold method identified an optimal threshold of 0.0006020622 m/s that provided the highest accuracy (i.e., maximized correct predictions). Using a lower or higher threshold would reduce the accuracy of detecting recumbent and moving animals (see Figure 3).

**Figure 4:**
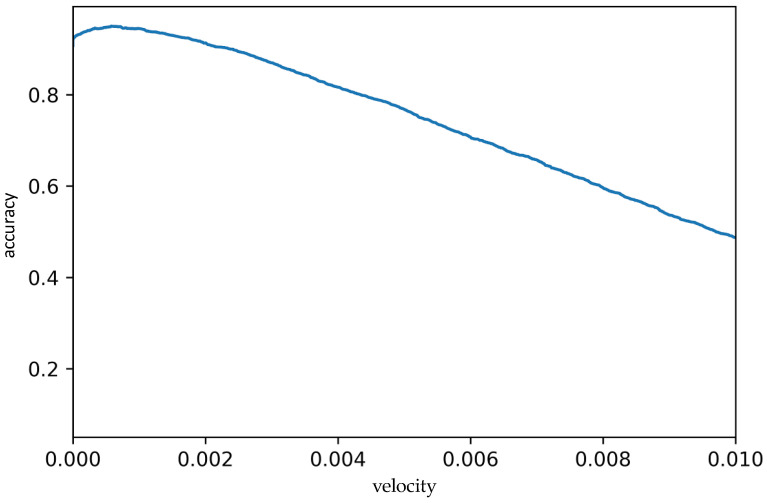
Classification accuracy of the automated method.

Figure 4 shows the classification accuracy according to the velocity threshold used to separate times when all pigs are recumbent from times when at least one pig is moving. The optimal threshold for correctly classifying whether all piglets are recumbent or at least one is standing yielded an accuracy of 94.1%, with a satisfactory sensitivity of 98.1% and an acceptable specificity of 60.8% for the used dataset. Table 4 shows the statistical results.

## 4. Discussion

Monitoring group recumbency in nonexperimental conditions on commercial farms is challenging and is often not possible for farmers. Employing methods to automatically detect images showing group recumbency can help to improve farm management on various levels. The use of camera systems for pig and farrow management is diverse and has been evaluated in previous works. A systematic review summarized the variety of possible applications of such systems in research and in practical use [34]. AI-supported systems can facilitate a wide range of identification, from behavioral detection to tracking of individual animals and disease diagnosis. This research subject has already been addressed by various research groups investigating the analysis and evaluation of imaging procedures for monitoring pigs [22,35]. The methodological approaches are diverse, but no reliable and uniform approach has been established thus far. Other authors have focused on recumbent pigs [22,36]. However, they used manual verification that the animals are recumbent, while our method allows automated detection of those pictures. In contrast to other studies, the present study developed a method for automatically detecting images containing all animals in a recumbent position. Our work outlines a suitable method for addressing challenges in which individual observation by artificial intelligence is made difficult by overlap, especially useful for piglets. The image data generated for our study can be used in a variety of ways. They can help farmers make decisions regarding animal welfare and provide a new perspective on previously overlooked issues.

In this analysis, the recumbent behavior of groups of weaners was investigated. The study revealed the overall good performance of this method, with a high accuracy of 94.1%. However, a high proportion of the collected data was not suitable for further analysis due to challenges regarding camera use on commercial farms. The different number of images per pen stems from the variation in the soiling of camera lenses over the pens during the test period. This had different causes but was mainly due to dust and flies. By calculating the blur for each image, different numbers of images per pen were removed from the dataset (9634 images at the beginning/3960 after adjustment). Filtering the data by an artificial neural network enabled fast data cleaning. That could be detected directly by the AI-supported camera system in the future to enable camera cleaning on-site. To generate a higher number of usable images, further investigations were made with a different type of camera. Bullet cameras tend to become less obscured under practical conditions than the dome cameras used in this experiment. However, this circumstance not only led to a limitation of the usable data but also provided insights for future research, especially regarding the practical use of different types of cameras. In farming practice, the usability of cameras is important. In addition to dirt obscuring the lens, there are other handling pitfalls, such as cleaning intervals, internet connectivity, and farmer acceptance of monitoring systems. Another important point is the data itself. Since the velocity values were very small, it was difficult to perform statistical analysis without converting the data. Another limitation is that when pigs lay on top of each other and one was in a half-standing position, the image was still coded as group recumbency. The results must also be interpreted with the caveat that pictures with recumbent pigs are underrepresented in the overall dataset, even though the pictures were selected at random. Future studies need to validate the method used here with a larger and more balanced dataset and test it in other barns. This study represents a preliminary evaluation of the methodology and provides evidence that this approach can be used for piglet assessments.

The overall goal of the present study was to identify only groups in which all weaners were recumbent. Among the images containing only recumbent pigs, 60.7% were correctly identified, which reflects an acceptable number of images. This system can thus provide farmers with containing only recumbent animals. Using the optimal threshold, the system misclassified over one-third of the images in which all weaners were recumbent as containing standing or moving piglets. However, the aim was to provide images in which all animals are recumbent. Therefore, the sensitivity of 97.8% is a satisfactory result to prevent false positives. Consequently, the system provides farmers with only pictures in which all animals are recumbent. From a management point of view, targeted detection of piglets exhibiting group recumbency is essential. The automated provision of such image data can enable farmers to evaluate resting behavior from various aspects. Thus, through visual observation, the farmer can analyze resting behavior in relation to the barn temperature and respond accordingly when behavioral indicators indicate that the environment is too warm or too cold. Such responses can improve the management system and thus the performance of the weaners [37,38]. Furthermore, by detecting these images, conspicuous alterations of resting behavior by individual animals in the group can be identified, for example, as indicators of possible disease [39,40] or of group exclusion of individual animals. However, this ability was not the subject of this investigation. In addition, this automated method has the potential to enable further automation of barn temperature conditions, health, and animal welfare measures through the correct identification of image data. The correct detection of images can be used for rearing analyses to perform similar investigations and techniques as those carried out in pig fattening. Additionally, the use of the provided images can improve farm management, as the farmer can interpret the number of group-recumbency periods. A uniform group recumbency period can be used as an indicator of well-being, conformity, and homogenous growth (which is economically relevant). Furthermore, group recumbency (and possibly the length of the recumbent phase) can be used to draw conclusions about the barn conditions. This can provide an economic advantage. The circadian rhythm of pigs also reflects whether the animals are doing well, especially in the weaning phase. In addition to the management aspects for the farmer, the provision of images can be used as a tool for health management. Assessment of the conditions in which group recumbency is exhibited can inform disease monitoring. Given the presence of diseases such as African swine fever, this approach is of great benefit to other parts of the value chain, such as veterinarians. From the point of view of health management, deviations in the behavior of individual animals can also be observed, and depending on the indication, targeted treatments can be carried out.

Various studies have investigated the use of technology to monitor pigs and weaners; these studies have had objectives ranging from health to animal well-being [41,42,43]. Our methodological approach enables the automatic detection of images in which all weaned pigs are recumbent, which can be used as a farm management tool. On the one hand, it represents an implementable solution for improved farm management using a system with object tracking. On the other hand, the automated detection of group recumbency in piglets is crucial input for further automation through artificial intelligence. The technique should be evaluated and compared with different camera systems. However, more images and data from different commercial operations are needed in the future.

## 5. Conclusions

In conclusion, the novel automated methodology developed in this study successfully detected group recumbency in piglets. These data were collected on farms; thus, this system can be used in practical farm management. More than 9000 images and associated velocity values were evaluated. Considering the abovementioned limitation of the dataset, the performance of our method showed high accuracy and sensitivity as well as acceptable specificity. Thus, this method could be used and marketed as a tool to improve farm management. This improvement not only assists farmers but can also others across the value chain as well as stakeholders. However, further studies and application of the generated images in automation scenarios are needed to enable widespread use and further exploitation of the data.

## Figures and Tables

**Table 1:** Sample Ethogram (List of Behaviors).

Behavior	Code	Behavior Description
Recumbent	0	Every animal in the group is recumbent (lying in a sternal or lateral position)
Standing	1	At least one animal in the group is not recumbent (i.e., an animal is standing or sitting).

**Table 2:** Performance criteria for the threshold method.

Performance Criteria for the Threshold Method		
Sensitivity (%)	$\frac{TP}{TP+FN}$	TP = true positive (standing position labeled as standing position) FP = false positive (recumbent position labeled as standing position) TN = true negative (recumbent position labeled as recumbent position) FN = false negative (standing position labeled as recumbent position)
Specificity (%)	$\frac{TN}{TN+FP}$	
Accuracy (%)	$\frac{TP+TN}{TP+FP+TN+FN}$	

**Table 3:** Number of images labeled as containing some standing or all recumbent pigs per pen.

	Number of Images	
Pen	Standing (1)	All Recumbent (0)
1	408	179
2	235	50
3	371	22
4	384	68
5	345	53
6	341	3
7	360	15
8	377	5
9	380	11
10	348	5
Total	3549	411

**Table 4:** Evaluation of the classification method.

Results of the Applied Optimal Threshold	
Sensitivity (%)	97.8%
Specificity (%)	60.8%
Accuracy (%)	94.1%

## Data Availability

The datasets analyzed during the current study are available from the corresponding author on reasonable request.

## References

[1] Yang Q., Xiao D. (2020). A review of video-based pig behavior recognition. Appl. Anim. Behav. Sci..

[2] Nasirahmadi A., Sturm B., Olsson A.-C., Jeppsson K.-H., Müller S., Edwards S., Hensel O. (2019). Automatic scoring of lateral and sternal lying posture in grouped pigs using image processing and Support Vector Machine. Comput. Electron. Agric..

[3] Riekert M., Klein A., Adrion F., Hoffmann C., Gallmann E. (2020). Automatically detecting pig position and posture by 2D camera imaging and deep learning. Comput. Electron. Agric..

[4] Xin H. (1999). Assessing Swine Thermal Comfort by Image Analysis of Postural Behaviors. J. Anim. Sci..

[5] Buayai P., Piewthongngam K., Leung C.K., Saikaew K.R. (2019). Semi-Automatic Pig Weight Estimation Using Digital Image Analysis. Appl. Eng. Agric..

[6] Liu D., Oczak M., Maschat K., Baumgartner J., Pletzer B., He D., Norton T. (2020). A computer vision-based method for spatial-temporal action recognition of tail-biting behaviour in group-housed pigs. Biosyst. Eng..

[7] Bateson M., Martin P. (2021). Measuring Behaviour: An Introductory Guide.

[8] Donát P. (1991). Measuring behaviour: The tools and the strategies. Neurosci. Biobehav. Rev..

[9] Lehner P.N. (1992). Sampling Methods in Behavior Research. Poult. Sci..

[10] Mullan S., Browne W.J., Edwards S.A., Butterworth A., Whay H.R., Main D.C.J. (2009). The effect of sampling strategy on the estimated prevalence of welfare outcome measures on finishing pig farms. Appl. Anim. Behav. Sci..

[11] Enders R.K., Carpenter C.R. (1934). A Field Study of the Behavior and Social Relations of the Howling Monkeys. J. Mammal..

[12] Schneirla T.C. (1950). The relationship between observation and experimentation in the field study of behavior. Ann. N. Y. Acad. Sci..

[13] Altmann J. (1974). Observational study of behavior: Sampling methods. Behaviour.

[14] Götz S., Raoult C.M.C., Reiter K., Wensch-Dorendorf M., von Borell E. (2022). Lying, Feeding and Activity Preference of Weaned Piglets for LED-Illuminated vs. Dark Pen Compartments. Animals.

[15] Marx D. (1991). Beurteilungskriterien für artgerechte Tierhaltung am Beispiel der Schweineaufzucht. Bau. Für Die Landwirtsch.

[16] Hörning B., Raskopf S., Simantke C. (1992). Artgemäße Schweinehaltung Grundlagen und Beispiele aus der Praxis.

[17] Mayer C., Hillmann E., Schrader L. (2006). Verhalten, Haltung und Bewertung von Haltungssystemen. Schweinezucht und Schweinefleischerzeugung: Empfehlungen für die Praxis.

[18] Opderbeck S., Keßler B., Gordillio W., Schrade H., Piepho H.-P., Gallmann E. (2020). Influence of A Cooled, Solid Lying Area on the Pen Fouling and Lying Behavior of Fattening Pigs. Agriculture.

[19] Scriba M., Wechsler B. (2021). Verhaltensindikatoren und physiologische Indikatoren für Hitzestress bei Mastschweinen. Agrar. Schweiz.

[20] Li D., Zhang K., Li Z., Chen Y. (2020). A Spatiotemporal Convolutional Network for Multi-Behavior Recognition of Pigs. Sensors.

[21] Huynh T., Aarnink A., Gerrits W., Heetkamp M., Canh T., Spoolder H., Kemp B., Verstegen M. (2005). Thermal behaviour of growing pigs in response to high temperature and humidity. Appl. Anim. Behav. Sci..

[22] Nasirahmadi A., Richter U., Hensel O., Edwards S., Sturm B. (2015). Using machine vision for investigation of changes in pig group lying patterns. Comput. Electron. Agric..

[23] Nasirahmadi A., Edwards S.A., Sturm B. (2017). Implementation of machine vision for detecting behaviour of cattle and pigs. Livest. Sci..

[24] Shao B., Xin H. (2008). A real-time computer vision assessment and control of thermal comfort for group-housed pigs. Comput. Electron. Agric..

[25] Shao J., Xin H., Harmon J.D. (1998). Comparison of image feature extraction for classification of swine thermal comfort behavior. Comput. Electron. Agric..

[26] Lao F., Brown-Brandl T., Stinn J.P., Liu K., Teng G., Xin H. (2016). Automatic recognition of lactating sow behaviors through depth image processing. Comput. Electron. Agric..

[27] Kemper N., Edwards S. (2021). Optimising pig welfare at the weaning and nursery stage. Understanding the Behaviour and Improving the Welfare of Pigs.

[28] Yilmaz A., Javed O., Shah M. (2006). Object tracking: A survey. ACM Comput. Surv..

[29] Krogh A. (2008). What are artificial neural networks?. Nat. Biotechnol..

[30] Ahrendt P., Gregersen T., Karstoft H. (2011). Development of a real-time computer vision system for tracking loose-housed pigs. Comput. Electron. Agric..

[31] He H., Qiao Y., Li X., Chen C., Zhang X. (2021). Optimization on multi-object tracking and segmentation in pigs’ weight measurement. Comput. Electron. Agric..

[32] Matthews S.G., Miller A.L., Plötz T., Kyriazakis I. (2017). Automated tracking to measure behavioural changes in pigs for health and welfare monitoring. Sci. Rep..

[33] Kuhn M. (2008). Building Predictive Models in R Using the caret Package. J. Stat. Softw..

[34] Wang S., Jiang H., Qiao Y., Jiang S., Lin H., Sun Q. (2022). The Research Progress of Vision-Based Artificial Intelligence in Smart Pig Farming. Sensors.

[35] Cook N.J., Bench C.J., Liu T., Chabot B., Schaefer A.L. (2018). The automated analysis of clustering behaviour of piglets from thermal images in response to immune challenge by vaccination. Animal.

[36] Nasirahmadi A., Hensel O., Edwards S.A., Sturm B. (2017). A new approach for categorizing pig lying behaviour based on a Delaunay triangulation method. Animal.

[37] Hoha G.V., Costachescu E., Nica A., Dunea I.B., Pasarin B. (2013). The influence of microclimates conditions on production performance in pigs. Lucr. Ştiinţifice Ser Zooteh.

[38] Le Dividich J. (1981). Effects of environmental temperature on the growth rates of early-weaned piglets. Livest. Prod. Sci..

[39] Le Dividich J., Herpin P. (1994). Effects of climatic conditions on the performance, metabolism and health status of weaned piglets: A review. Livest. Prod. Sci..

[40] Sutherland M.A., Niekamp S.R., Johnson R.W., Van Alstine W.G., Salak-Johnson J.L. (2007). Heat and social rank impact behavior and physiology of PRRS-virus-infected pigs. Physiol. Behav..

[41] Chen C., Zhu W., Steibel J., Siegford J., Han J., Norton T. (2020). Recognition of feeding behaviour of pigs and determination of feeding time of each pig by a video-based deep learning method. Comput. Electron. Agric..

[42] Fernandes A.F.A., Dórea J.R.R., Fitzgerald R., Herring W., Rosa G.J.M. (2019). A novel automated system to acquire biometric and morphological measurements and predict body weight of pigs via 3D computer vision. J. Anim. Sci..

[43] Yik S., Benjamin M., Lavagnino M., Morris D. DIAT (Depth-Infrared Image Annotation Transfer) for Training a Depth-Based Pig-Pose Detector. Proceedings of the 2020 IEEE/RSJ International Conference on Intelligent Robots and Systems (IROS).

